# Acute effects of different resistance training intensities on creative thinking in university students

**DOI:** 10.3389/fspor.2026.1807186

**Published:** 2026-05-01

**Authors:** Jun Zhou, Yiqing Xie, Yemin Han

**Affiliations:** 1 School of Physical Education, Shanghai University of Sport, Shanghai, China; 2 Sport, Exercise and Health Sciences, Loughborough University, Loughborough, United Kingdom

**Keywords:** creative thinking, divergent thinking, exercise intensity, resistance exercise, university students

## Abstract

**Background:**

Physical activity has been shown to enhance creative thinking; however, existing research has primarily focused on aerobic exercise, and evidence regarding the acute effects of different resistance training intensities on creative thinking remains limited.

**Methods:**

Using a within-subjects design, 30 university students completed resistance training sessions at 40%, 60%, and 80% one-repetition maximum (1RM). Creative performance was assessed immediately after exercise using the Alternate Uses Test (AUT), measuring fluency, flexibility, and novelty.

**Results:**

Exercise intensity significantly influenced all three creativity indicators. Moderate-intensity resistance training (60% 1RM) resulted in significantly greater fluency, flexibility, and novelty scores compared with both low- (40% 1RM) and high-intensity (80% 1RM) conditions.

**Conclusions:**

Post-exercise creative performance differed across the three tested resistance-training intensities, with moderate loads showing the most favorable pattern. These findings suggest that, among the three tested conditions, moderate-intensity resistance training was associated with the highest post-exercise creative performance in university students.

## 1 Introduction

Physical activity promotes mental health and enhances cognitive functions such as executive control, attention, and memory processes (1–7). A growing body of evidence indicates that regular engagement in physical activity provides multidimensional benefits for students that extend beyond physical health, including improvements in learning efficiency, psychological well-being, and resilience (8–13). Exercise is widely recognized as a cornerstone of overall health, contributing to cardiovascular fitness (14), weight management (15), immune function (16), and positive self-perception, including increased confidence and improved body image (17–19).

In recent years, creative thinking has been increasingly emphasized as a core competency in higher education due to its critical role in learning, problem solving, and future career development (20–22). Creative cognition involves a dynamic interaction among executive control, attention regulation, and memory processes, enabling individuals to generate novel and appropriate ideas (23). Consequently, researchers have begun to explore modifiable lifestyle factors that may enhance creative performance (24, 25).

Accumulating evidence suggests that physical activity represents a promising intervention for improving creative thinking. A recent multilevel meta-analysis demonstrated that both acute and chronic physical activity exert medium-sized positive effects on creative ideation, including fluency, flexibility, and novelty (22). Experimental studies further indicate that acute aerobic exercise can enhance divergent thinking in children and adults, while structured exercise programs have been associated with improvements in cognitive flexibility and creativity among university students (26–32). These findings imply that even brief bouts of physical activity may induce measurable changes in creative cognition.

From a mechanistic perspective, physical activity increases cerebral blood flow and stimulates exercise-induced neuroplasticity. Moreover, exercise promotes the release of neurotrophic factors such as brain-derived neurotrophic factor (BDNF), insulin-like growth factor-1 (IGF-1), and vascular endothelial growth factor (VEGF), which collectively support synaptic plasticity and higher-order cognitive processing (21). Through these pathways, physical activity may indirectly facilitate creativity by strengthening neural substrates underlying executive function and associative thinking.

Although prior research has predominantly focused on aerobic exercise (26–31, 33, 34), emerging evidence indicates that resistance training may also confer cognitive benefits (35–40). Resistance exercise has been shown to induce central nervous system adaptations and improve executive functioning, even following a single training session (41, 42). Compared with aerobic modalities, resistance training involves greater movement complexity, sensorimotor integration, and task-specific attentional demands, potentially engaging distinct neural pathways relevant to creative cognition (43–46). In addition, resistance training is highly accessible and time-efficient, making it a practical strategy for cognitive enhancement in educational contexts.

Despite these promising findings, important gaps remain (32). First, creativity-related exercise research has largely centered on aerobic activity, whereas the effects of resistance training on creative thinking remain underexplored, and no previous study has directly examined this association. Second, although exercise intensity is recognized as a critical moderator of cognitive outcomes, few studies have systematically examined how different resistance training intensities influence creative performance. Existing evidence suggests that moderate-intensity exercise may optimize cognitive benefits; however, this hypothesis has rarely been tested within resistance training paradigms.

Therefore, the present study employed a within-subjects design to investigate the acute effects of three resistance training intensities (40%, 60%, and 80%1RM) on creative thinking in university students. Creative performance was assessed using the AUT, capturing fluency, flexibility, and novelty. Based on previous theoretical frameworks and empirical findings, we hypothesized that (1) resistance training intensity would significantly influence creative thinking performance, and (2) moderate-intensity resistance exercise (60%1RM) would be associated with higher post-exercise creative ideation scores than the low- and high-intensity conditions.

## 2 Materials and methods

### 2.1 Participants

*A priori* sample size estimation was conducted using G*Power software for a one-way repeated-measures ANOVA. Because no prior study had directly examined the acute effects of different resistance-training intensities on creative thinking, the expected effect size was set at f = 0.25, corresponding to a medium effect according to Cohen's convention and representing a reasonable planning assumption. With *α* = 0.05 and powe*r* = 0.80, the analysis indicated that a minimum of 28 participants was required. Thirty university students (18 males and 12 females; mean age = 20.98 years, range = 18–24 years) were recruited for this study. Regarding resistance training experience, four participants had one year or less, four had more than one year but no more than three years, one had more than three years but no more than five years, and the remaining 21 participants had no prior resistance training experience. All participants were right-handed. The study protocol was approved by the Ethics Committee of Shanghai University of Sport (Approval No. 102772025RT044). All participants provided written informed consent prior to participation.

### 2.2 Experimental design

**Figure 1:**
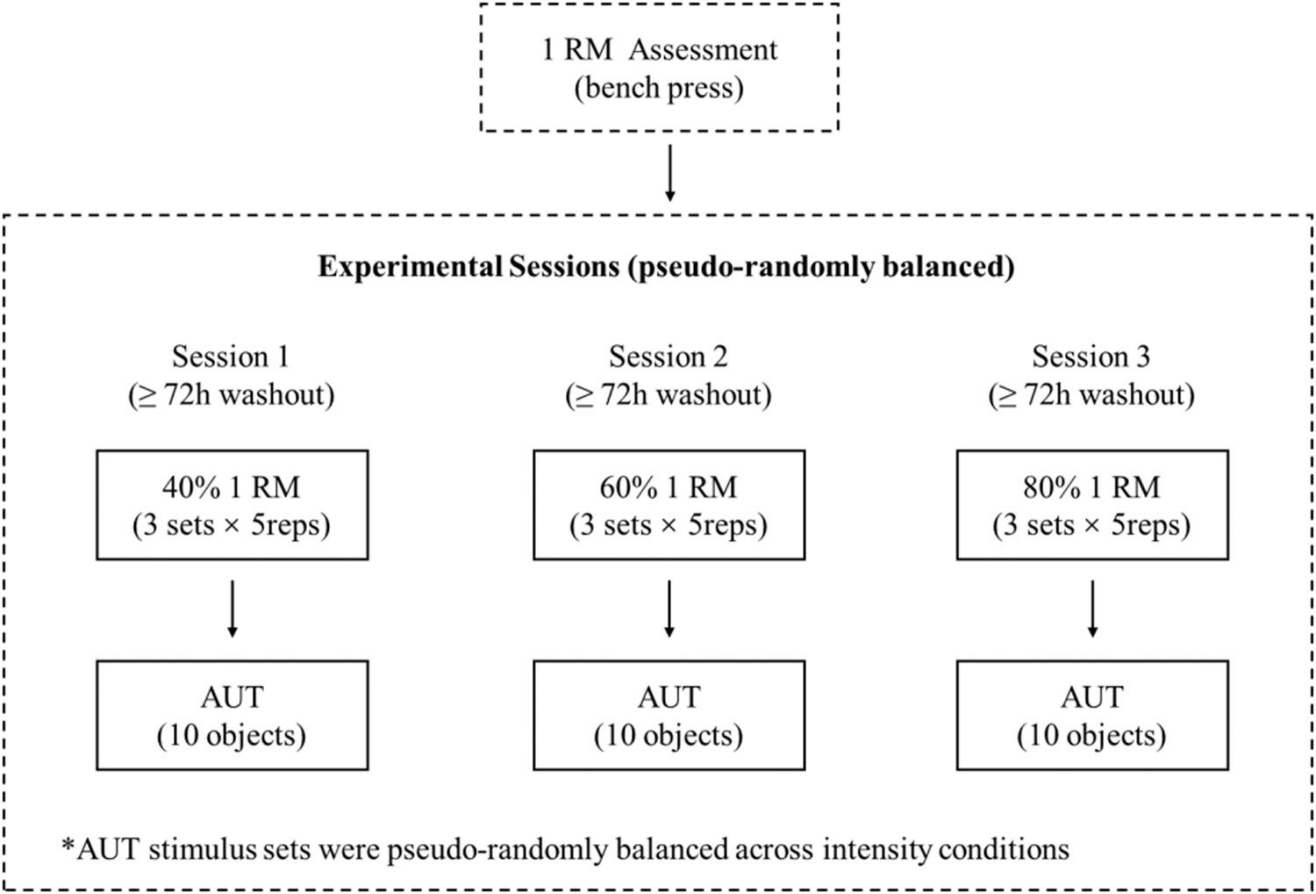
Schematic representation of the experimental procedure.

A single-factor within-subjects design was employed to examine the effects of three resistance training intensities (low, moderate, and high) on creative thinking assessed by the AUT. Each participant completed all three intensity conditions. To avoid the influence of fatigue and order effects, the order of the three intensity conditions was pseudo-randomly balanced across participants, and each participant was randomly assigned to one of the six possible order sequences. Due to the pseudo-randomization procedure, there were five participants in each sequence, effectively controlling for potential order effects. A minimum washout period of 72 h and no more than one week separated consecutive sessions. Immediately after each training session, participants were escorted to a quiet room to complete the AUT. The specific experimental procedure is shown in Figure 1.

Participants first completed a one-repetition maximum (1RM) assessment for the bench press. Subsequently, they attended three experimental sessions in a counterbalanced order, with a minimum interval of 72 h between sessions. In each session, participants performed a resistance training protocol consisting of three sets of five repetitions at one of three intensities (40% 1RM, 60% 1RM, or 80% 1RM), immediately followed by the Alternate Uses Test (AUT). The three sets of AUT stimuli were counterbalanced across intensity conditions.

### 2.3 Resistance training protocol

#### 2.3.1 One-repetition maximum (1RM) assessment

All testing and training sessions were conducted at the Strength and Conditioning Research Center (Shanghai, China). The laboratory was temperature-controlled (20–24 °C), well-lit, and isolated from external noise to minimize distractions. Participants were tested individually between 9:00 AM and 12:00 PM to control for circadian variations. The participants arrived and confirmed that they had complied with the requirements before the experiment (such as not engaging in strenuous exercise within 24 h, and not consuming caffeine or alcohol). All resistance training was performed using a Smith machine (Lipper, Nantong, China).

During the initial familiarization session, participants completed a standardized 10 min warm-up, including jogging, dynamic stretching, upper-limb and shoulder mobility exercises, and one explosive set of five repetitions using a 17 kg external load (the unloaded Smith machine bar). After a 3 min rest, bench press 1RM was determined following a standard incremental protocol. The initial load was set at 17 kg and progressively increased by 10 kg increments. Thereafter, load was increased in steps of 5–1 kg until the actual 1RM was directly determined by completing a single maximal lift. Two trained spotters were positioned on each side of the barbell to ensure safety and provide verbal encouragement. Participants performed the bench press using the standard five-point body contact technique (head, upper back, and buttocks firmly on the bench, with both feet flat on the floor). Grip width was self-selected and kept constant across all sessions. Each repetition began with elbows fully extended. Participants were instructed to perform both the eccentric and concentric phases as fast as possible. The barbell was lowered to the sternum level, and the concentric phase ended when the elbows reached full extension.

#### 2.3.2 Resistance training intensity conditions

In the subsequent three experimental sessions, participants completed resistance training at three intensities (40%, 60%, and 80% 1RM), using the 1RM value obtained during the initial assessment session. For each condition, participants performed three sets of five repetitions, with 3 min rest between sets. Physiological indicators such as heart rate or rating of perceived exertion (RPE) were not assessed during the exercise sessions.

### 2.4 Creative thinking assessment

#### 2.4.1 Alternate uses test (AUT) procedure

Creative thinking was assessed using a modified version of the AUT based on Guilford's classical creativity paradigm (47). Participants were instructed to generate as many unconventional uses as possible for common objects (e.g., pencil). A total of 30 stimulus objects were evenly divided into three task sets (10 objects per condition). The task sets were pseudo-randomly balanced across exercise conditions.

Prior to testing, participants completed practice trials to familiarize themselves with the procedure. Participants were instructed to generate as many uses as possible for each object within one minute, without an explicit prompt to be creative. The instruction stated: “Next, some item names will be displayed one by one on the screen. Please list as many uses as possible for the object presented on the screen within one minute. The uses can be either common or uncommon.” During testing, stimulus words (e.g., brick, newspaper, rope) were presented individually on a computer screen. Each trial began with a 500 ms fixation cross (“+”), followed by presentation of an object name for 1 min. Participants were instructed to verbally generate as many unconventional uses as possible during this period. Each condition consisted of 10 trials.

#### 2.4.2 Scoring of the AUT

All responses were transcribed verbatim and scored by five independent raters (mean age = 32.4 years, range = 26–41; 3 females, 2 males) who were blinded to the experimental conditions. All raters held a master's degree or higher in sport science or psychology and had prior experience with divergent thinking assessment. Three dimensions of divergent thinking were assessed: fluency, flexibility, and novelty.

Fluency was defined as the total number of valid uses generated per object. Invalid responses (e.g., irrelevant, incomprehensible, or repeated uses) were excluded. The fluency score for each participant under each condition was calculated as the mean number of valid uses across the 10 objects.

Flexibility was defined as the number of distinct conceptual categories of uses generated per object. The categorization scheme was developed based on a pilot dataset and adjusted iteratively by the five raters. For example, for the object “brick” uses such as “build a wall” and “build a house” were classified under the same category (“construction”), while “paperweight” and “doorstop” were classified under different categories (“weight” and “stopper” respectively). Each response was assigned to one category, and flexibility was calculated as the mean number of categories per object across the 10 stimuli.

Novelty was rated on a 5-point Likert scale (1 = “not at all original” to 5 = “very original”) based on the statistical infrequency and creative quality of each response. Raters were instructed to consider how uncommon the use was relative to the full dataset of responses. The novelty score for each participant was calculated as the mean rating across all valid responses. Prior to formal scoring, all five raters underwent a training session using a separate set of AUT responses (not included in the main dataset). During training, raters discussed and refined the category system for flexibility and calibrated their novelty ratings to ensure consistency. For the formal scoring, each rater independently scored all responses. Inter-rater reliability was assessed using Cronbach’ s *α*, yielding values of 0.99 for fluency, 0.90 for flexibility, and 0.76 for novelty. After independent scoring, the few cases with substantial discrepancies (e.g., >2 points difference on the 5-point novelty scale) were resolved through consensus discussion to obtain a single score for subsequent analysis.

## 3 Results

### 3.1 Fluency

**Figure 2:**
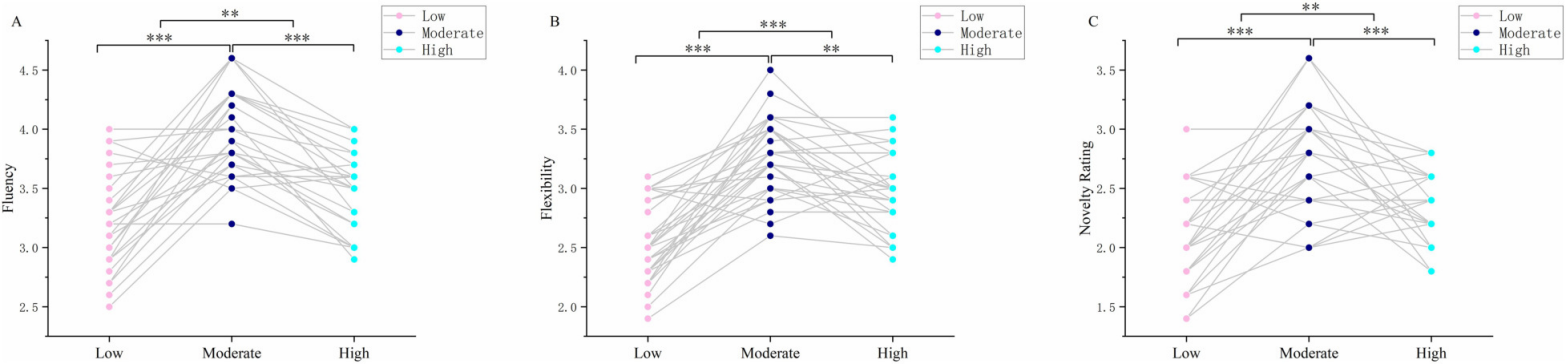
**Effects of different resistance training intensities on divergent thinking.** Fluency scores represent the mean number of responses generated per object under each training intensity **(A)**; flexibility scores represent the mean number of distinct categories of responses generated under each training intensity **(B)**; novelty scores represent the mean ratings of novelty by five raters using a 5-point scale (1 = “not at all original” to 5 = “very original”) under each training intensity **(C)** Error bars represent the standard error of the mean. ** *p* < 0.01, *** *p* < 0.001.

A one-way repeated-measures ANOVA revealed a significant main effect of exercise intensity on fluency, *F*(1.55, 45.04) = 41.50, *p* < 0.001, *η*_p_^2^ = 0.589. *post hoc* comparisons with Bonferroni adjustment indicated that fluency was significantly higher following moderate-intensity resistance exercise (60%1RM) compared with both low-intensity (40%1RM, *p* < 0.001) and high-intensity (80%1RM, *p* < 0.001) conditions. In addition, fluency under high-intensity exercise was significantly greater than under low-intensity exercise (*p* < 0.01). Fluency results are illustrated in Figure 2A.

### 3.2 Flexibility

A significant main effect of exercise intensity was also observed for flexibility, *F*(1.61, 46.67) = 18.32, *p* < 0.001, *η*_p_^2^ = 0.392. *post hoc* comparisons with Bonferroni adjustment indicated that flexibility following moderate-intensity exercise was significantly higher than that following both low-intensity (*p* < 0.001) and high-intensity (*p* < 0.01) exercise. In addition, flexibility under high-intensity exercise was significantly greater than under low-intensity exercise (*p* < 0.001). Flexibility results are illustrated in Figure 2B.

### 3.3 Novelty rating

Exercise intensity exerted a significant effect on novelty rating, *F*(1.63, 47.19) = 21.47, *p* < 0.001, *η*_p_^2^ = 0.426. *post hoc* comparisons with Bonferroni adjustment indicated that novelty ratings were significantly higher after moderate-intensity resistance training compared with both low-intensity (*p* < 0.001) and high-intensity (*p* < 0.001) conditions. Furthermore, novelty rating following high-intensity exercise was significantly greater than that following low-intensity exercise (*p* < 0.01). Novelty rating results are illustrated in Figure 2C.

### 3.4 Correlations among divergent thinking indicators

To examine the relationships among the three divergent thinking dimensions, Pearson correlation coefficients were calculated using the mean scores across all three intensity conditions. The results revealed a significant strong positive correlation between fluency and flexibility (*r* = 0.793, *p* < 0.001). However, no significant correlations were found between fluency and novelty (*r* = 0.129, *p* = 0.496), or between flexibility and novelty (*r* = –0.061, *p* = 0.750). These results suggest that, in the present study, fluency and flexibility reflect a shared underlying process, whereas novelty appears to be relatively independent from the other two dimensions.

## 4 Discussion

The present study examined the acute effects of different resistance training intensities on creative thinking in university students. The results revealed a significant effect of exercise intensity on fluency, flexibility, and novelty, with moderate-intensity resistance exercise (60% 1RM) yielding the highest post-exercise scores across all three creativity dimensions. These findings support our hypotheses and indicate that post-exercise creative performance differed across the three tested resistance-training intensities.

This pattern is broadly consistent with previous research suggesting that moderate-intensity physical activity may be associated with more favorable cognitive performance, including creativity. Prior meta-analyses have indicated that moderate-intensity exercise optimizes cognitive outcomes by balancing physiological arousal and cognitive processing (21, 22). Low-intensity exercise may fail to provide sufficient stimulation, while high-intensity exercise can induce fatigue and resource competition, which may impair cognitive performance. Our results extend this line of research by demonstrating that moderate-intensity resistance training promotes creative thinking more effectively than both low- and high-intensity conditions (26–31, 33, 34).

From a mechanistic perspective, creativity is believed to depend on the dynamic interaction of executive control, attentional regulation, and memory processes (21, 22). Physical activity has been shown to enhance these cognitive functions by increasing cerebral blood flow and inducing exercise-related neuroplasticity (3, 20). In particular, resistance exercise stimulates the release of neurotrophic factors such as brain-derived neurotrophic factor (BDNF), insulin-like growth factor-1 (IGF-1), and vascular endothelial growth factor (VEGF), which support synaptic plasticity and higher-order cognitive processing (21). Given these mechanisms, one possible explanation for the more favorable post-exercise creative performance observed under moderate-intensity resistance training is enhanced neural activation in brain regions related to executive function and associative thinking (41, 42). However, this interpretation remains speculative because no biomarkers were assessed in the present study. Compared with aerobic exercise, resistance training may place greater demands on motor coordination, sensorimotor integration, and task-specific attentional control, and may therefore engage somewhat different neural adaptations relevant to higher-order cognition (21, 43–46). These features may contribute to cognitive flexibility and novelty-related processes by involving brain networks associated with attention and motor planning.

In addition to the neurocognitive and physiological pathways discussed above, psychological mechanisms may also help explain the observed effects. In particular, positive affect and exercise-induced arousal have been proposed as potential pathways through which physical activity may facilitate creative ideation (22, 30). Affective changes following exercise may broaden attentional scope and promote cognitive flexibility, which could in turn support divergent thinking (22, 30). However, the current evidence remains limited, and the mediating role of positive affect has not yet been conclusively established (22, 34). Therefore, this interpretation should be considered tentative, especially in the context of resistance training. The mechanisms discussed above may not be specific to creativity-based performance alone, but may also apply to broader aspects of cognitive functioning. At the same time, creativity-based tasks such as the AUT may place greater demands on cognitive flexibility and associative processing. Future research should therefore include both creativity-based and non-creativity cognitive tasks to examine whether the effects of resistance training are specific or more general.

Notably, the correlational analysis in this study revealed that novelty was not significantly associated with either fluency or flexibility, suggesting that generating novel ideas may involve cognitive processes distinct from those underlying the mere quantity or categorical breadth of ideas. This finding aligns with previous research indicating that originality in divergent thinking tasks may rely more heavily on associative processes and cognitive flexibility under specific constraints (48), whereas fluency and flexibility may reflect more basic ideational productivity. Furthermore, the instructions for the Alternative Uses Test (AUT) used in this study did not explicitly emphasize creativity or originality, which may have led participants to prioritize fluency over novelty. While this procedural choice avoids potential demand characteristics, it also suggests that the observed effects on novelty should be interpreted with caution.

Despite these promising findings, there are several limitations to this study. First, the sample consisted of university students, which limits the generalizability of the findings to other age groups or populations. Future research should explore the effects of resistance training on creativity in different age groups, including children and older adults. Second, while this study focused on the acute effects of exercise, future studies should examine the long-term effects of resistance training on creative thinking, as well as the underlying neurobiological mechanisms. Third, we did not collect manipulation-check indicators such as rating of perceived exertion (RPE), heart rate, or other internal-load measures during the exercise sessions, and we also did not assess participants’ affective state. Therefore, we could not directly verify the separation of the three intensity conditions in perceived or physiological intensity, nor could we examine whether affective changes contributed to the observed differences in creative performance. Future studies should include both internal-load and affective measures. Fourth, the present sample was heterogeneous with respect to prior resistance training experience, with the majority of participants being inexperienced. Due to the limited sample size and unbalanced distribution across experience levels, we were unable to statistically examine whether training experience moderated the observed effects. Finally, the present study employed a post-exercise only design without a pre-exercise baseline or a non-exercise control condition. Therefore, the observed effects reflect differences among intensity conditions rather than absolute improvements from pre-exercise levels. Future studies should include baseline measurements and a control condition to better establish the causal effects of resistance training on creative thinking. In addition, the present study assessed creative performance only immediately after the resistance-training bout. Therefore, it remains unclear whether similar effects would be observed during exercise or after a longer post-exercise delay. Future research should also incorporate baseline measurements and control groups to better establish causal relationships, while examining the effects both during exercise and at different post-exercise delay intervals.

## 5 Conclusion

This study demonstrates that acute moderate-intensity resistance training (60% 1RM) is associated with superior creative thinking performance in university students, as reflected by higher fluency, flexibility, and novelty scores compared to low- and high-intensity conditions. These findings highlight the importance of exercise intensity in optimizing creativity and suggest that moderate-load resistance training may represent a promising direction for future research on exercise intensity and creative performance in university settings.

## Data Availability

The original contributions presented in the study are included in the article/Supplementary Material, further inquiries can be directed to the corresponding author.
